# Acknowledgement to Reviewers of *Journal of Functional Biomaterials* in 2019

**DOI:** 10.3390/jfb11010006

**Published:** 2020-01-20

**Authors:** Journal of Functional Biomaterials Editorial Office

**Affiliations:** MDPI, St. Alban-Anlage 66, 4052 Basel, Switzerland

The editorial team greatly appreciates the reviewers who have dedicated their considerable time and expertise to the journal’s rigorous editorial process over the past 12 months, regardless of whether the papers are finally published or not. In 2019, a total of 56 papers were published in the journal, with a median time to first decision of 18 days and a median time from submission to publication of 44 days. The editors would like to express their sincere gratitude to the following reviewers for their generous contribution in 2019:
Aguilar, Ludwig ErikLin, Tz-FengAlvarez-Lorenzo, CarmenLindberg, GabriellaAmbu, RitaMagyari, KlaraAmza, CatalinMajumder, PoulamiBaino, FrancescoMarrazzo, PasqualeBanas, JeffreyMarto, Carlos MiguelBanchelli, MartinaMeisel, Hans-JörgBarbeck, MikeMessias, AnaBeltrán, Ana M.Metzinger, LaurentBernardi, SaraMokhtari, SaharBoda, Sunil KumarMukherjee, SudipBouropoulos, NikolaosMulloy, BarbaraBoyer, Christen J.Muntimadugu, EameemaBruzell, EllenNam, Seung YunCacciotti, IlariaNandi, SaikatCasimiro, Maria HelenaNarayanan, GaneshChateigner, DanielNg, Wei LongChen, ZhitongNguyen, Ba Thuy LinhChereddy, KiranNicholson, John W.Chern, EdwardNisnevitch, MarinaChin, Kok YongOkunkova, Anna A.Ciapetti, GabrielaOtsuka, YutaCrisci, AlessandroPadmanabhan, JagannathCsapo, EditPagano, StefanoDaprile, GiuseppePaknahad, AliDiaz-Rodriguez, PatriciaPatterson, JenniferDinca, AnaPeng, ChaoDodero, VeronicaPerale, GiuseppeDonnermeyer, DavidPiluso, SusannaDorozhkin, Sergey V.Pogorielov, MaksymDutour Sikirić, MajaRabadán-Ros, RubénEglin, DavidRaspanti, MarioElder, SteveRíos-Carrasco, BlancaFernández-Arévalo, MercedesRizzolio, FlavioFigueiras, AnaRodríguez-Lorenzo, LuisFregnan, FedericaRodríguez-Lozano, Francisco JavierFröhlich, EleonoreRomán-Doval, RamónGabrić, DraganaSapudom, JiranuwatGarcia-Gonzales, CarlosScarano, AntonioGiudice, Giuseppe LoSchmidt, FranziskaGomez-Lazaro, MariaSefat, FarshidGrzech-Leśniak, KingaShen, GuofangGu, ChunjuShojaeiarani, JamilehGundapaneni, DineshSills, E ScottHama, SusumuSolitro, GiovanniHamon, MorganStrudwick, XantheHe, JingweiSutariya, VijaykumarHegedűs, CsabaTabata, YasuhikoHixon, Katherine R.Tallarico, MarcoHoshiba, TakashiTang, HouliangHsieh, Chih-ChenTing, Jeffrey M.Hu, YangTölli, HannaHuang, YugangTruong, Vi KhanhIbrahim, ToniTsai, Ang-ChenIllescas Montes, Rebeca IllescasTsuchiya, AkiraIncarnato, LoredanaTurco, GianlucaIordache, FlorinVilà, AnnaIseki, SachikoWalsh, Laurence J.Jammalamadaka, UdayabhanuWalsh, Pamela J.Joly-Duhamel, ChristineWang, XiaojuKazek-Kęsik, AlicjaWang, YuchenKeller, BrandisWelshhans, KristyKengelbach-Weigand, AnnikaWitek, LukaszKevadiya, BhaveshYadavalli, Nataraja SekharKijenska, EwaYang, TaoKolmas, JoannaYazdi, ImanKoltz, Michael T.Yu, LuKoutavarapu, RavindranadhZeitani, JacobKurečič, ManjaZhang, YiLee, MiyoungZhou, YubinLi, Ming-ChiaZhu, LiLin, Maohua

